# Rates of Assessment of Social Media Use in Psychiatric Interviews Prior to and During COVID-19: Needs Assessment Survey

**DOI:** 10.2196/28495

**Published:** 2021-09-14

**Authors:** Shiri Raphaely, Simon B Goldberg, Megan Moreno, Zachary Stowe

**Affiliations:** 1 Department of Psychiatry University of Wisconsin Madison, WI United States; 2 Department of Counseling Psychology University of Wisconsin Madison, WI United States; 3 Department of Pediatrics University of Wisconsin Madison, WI United States

**Keywords:** social media, screentime, problematic Internet use, psychiatric interview, psychiatric training, COVID-19, residency, training, survey, psychiatry, evaluation, quarantine

## Abstract

**Background:**

Current research suggests that there is a nuanced relationship between mental well-being and social media. Social media offers opportunities for empowerment, information, and connection while also showing links with depression, high-risk behavior, and harassment. As this medium rapidly integrates into interpersonal interactions, incorporation of social media assessment into the psychiatric evaluation warrants attention. Furthermore, the COVID-19 pandemic and containment measures (ie, social distancing) led to increased dependence on social media, allowing an opportunity to assess the adaptation of psychiatric interviews in response to sociocultural changes.

**Objective:**

The first aim of this study was to evaluate if general psychiatry residents and child and adolescent psychiatry fellows assessed social media use as part of the clinical interview. Second, the study examined whether changes were made to the social media assessment in response to known increase of social media use secondary to social distancing measures during the COVID-19 pandemic.

**Methods:**

As part of a quality improvement project, the authors surveyed general psychiatry residents and child psychiatry fellows in a university-based training program (n=21) about their assessment of social media use in patient evaluations. Soon after the survey closed, “stay-at-home” orders related to the COVID-19 pandemic began. A subsequent survey was sent out with the same questions to evaluate if residents and fellows altered their interview practices in response to the dramatic sociocultural changes (n=20).

**Results:**

Pre-COVID-19 pandemic survey results found that 10% (2/21) of respondents incorporated social media questions in patient evaluations. In a follow-up survey after the onset of the pandemic, 20% (4/20) of respondents included any assessment of social media use. Among the 15 participants who completed both surveys, there was a nonsignificant increase in the likelihood of asking about social media use (2/15, 13% vs 4/15, 27%, for pre- and during COVID-19, respectively; McNemar χ^2^_1_=0.25, *P*=.617, Cohen *d*=0.33).

**Conclusions:**

These small survey results raise important questions relevant to the training of residents and fellows in psychiatry. The findings suggest that the assessment of social media use is a neglected component of the psychiatric interview by trainees. The burgeoning use and diversity of social media engagement warrant scrutiny with respect to how this is addressed in interview training. Additionally, given minimal adaptation of the interview in the midst of a pandemic, these findings imply an opportunity for improving psychiatric training that incorporates adapting clinical interviews to sociocultural change.

## Introduction

Social media can be defined as computer-mediated technology that allows individuals to use online profiles to share user-generated content as well as network and communicate within the platform [1]. In the United States, 90% of adolescents [2] and 72% of adults report using at least one social media platform [3], and it is rapidly becoming a significant component, if not the leading one, in many interpersonal interactions.

Social connection is a long-investigated component of mental well-being. Social isolation and loneliness are associated with negative mental health outcomes including depression and suicidality [4], and in turn, symptoms of psychiatric illnesses can make it more difficult to establish and sustain close relationships [5]. Social support has been shown to bolster resilience to stress, improve functionality in the setting of trauma-related disorders, and decrease the rate of medical comorbidities [6]. As social media becomes integrated into the fabric of human connection, it has great potential to influence mental well-being, and incorporation of social media assessment into the psychiatric evaluation as part of a complete history warrants attention.

Rather than being uniformly negative or positive, the psychological impact of social media is nuanced [7]. Benefits include easier access to new information and more opportunities for interaction with support networks that otherwise may not be as easily accessible [8]. This is particularly significant for patients with disabilities, those who identify with a marginalized group, and those who live in more geographically isolated areas [7]. Social media platforms can be spaces for positive support around healthy behaviors including smoking cessation, meditation, exercise, and nutrition [7].

In contrast, research indicates that problematic internet use and gaming could reflect symptomology of addiction, with 50% of adolescents and 27% of adults stating they feel “addicted” to their device [9]. Social media use also creates new arenas for sexual exploitation, harassment, and bullying as well as for exposure and access to potentially harmful behaviors including drugs, alcohol, and higher-risk sexual behavior [7].

The specific impact of social media use seems to be influenced by how and how much individuals engage. For example, adolescents who spend more than 3 hours per day using social media may be at heightened risk for mental health problems, and those who actively post and share content report increased satisfaction than those who engage more passively [10,11]. A survey of young adults indicated that, while social media use itself was a poor predictor of mental health outcomes, “vaguebooking,” a style of post offering little information but written to elicit concern, is correlated with increased risk for suicidal ideation [12].

Despite evidence linking well-being with some social media usage patterns, there are not official guidelines on screening for social media use within psychiatry. In January 2020, the University of Wisconsin psychiatry residency program held both a journal club and a Grand Rounds on the topic of social media and mental health. While those in attendance clearly recognized the relevance of the topic, we questioned whether our clinical training reflected this perspective. We developed a quality improvement project to examine this, starting with a survey of current clinic practice.

Soon after this survey was closed, the COVID-19 pandemic began, leading to a significant increase in social isolation and increased use of social media for connection and communication [13]. A recent article examining social media use in Wuhan, China during lockdown due to COVID-19 indicated that there were here higher rates of anxiety and depression among individuals with more social media exposure [14]. The unique circumstances instigated by the pandemic prompted further investigation into whether the frequency of assessing social media use during psychiatric interviews increased in response to this global shift in communication practices.

## Methods

The pre-COVID-19 survey was developed as part of a needs assessment for a quality improvement project that aimed to identify rates of assessment of social media by residents during psychiatric interviews. The survey included 5 items (see Multimedia Appendix 1). Participants rated their own knowledge about social media use and mental health on a 3-point scale: very, somewhat, and not familiar (item 1). Participants indicated whether they routinely asked patients about their social media use (“Yes,” “No,” “only inpatient,” “only emergency room”; item 2). Participants then indicated whether they had clinical cases where they found social media use to be of benefit (item 3) or harm (item 4) to their patients. Lastly, participants indicated whether they would find a brief interview guide useful for their clinical practice (item 5).

The pre-COVID-19 survey was sent by email to a listserv including residents and child and adolescent fellows in the Department of Psychiatry at the University of Wisconsin. This listserv included 42 individuals. Of note, residents begin evaluating child and adolescent patients halfway through their intern year, thus all participants have exposure to patients of all ages. Respondents were entered into a random drawing to receive a gift card as thanks for participation. The pre-COVID-19 survey was open from February 7, 2020 through March 5, 2020.

On March 21, 2020, in response to the COVID-19 pandemic, the Wisconsin governor passed a “safer at home order.” Businesses, schools, and parks were closed, and people were confined to their homes. A second survey was sent out to the same listserv. This “during COVID-19 survey” reassessed participants’ frequency of asking about social media use since the safer at home order and their perception of benefit or harm of social media use (ie, items 2, 3, and 4 from the pre-COVID-19 survey). The during COVID-19 survey was opened on May 7, 2020 and closed on June 1, 2020.

We focused primarily on descriptive statistics for data collected pre- and during COVID-19. However, as pre- and during COVID-19 data were available for a subset of participants, we also conducted exploratory McNemar tests to evaluate whether responses changed significantly. Given the small sample size and likelihood that tests were underpowered, we included Cohen *d* effect sizes to allow interpretation of the magnitude of changes pre- and during COVID-19.

## Results

The response rate for the pre-COVID-19 survey was 50% (21/42) and for the during COVID survey was 48% (20/42); 15 participants completed the survey at both time points. The majority of participants (17/21, 81%) indicated they were “somewhat familiar” with the impact of social media on mental health. Most respondents reported having noted clinical case(s) in which social media was of benefit (13/21, 62%) as well as cases in which social media was hazardous (17/21, 81%). However, a minority of residents and fellows (2/21, 10%) routinely asked patients about their social media use pre-COVID-19. All participants noted a brief interview guide for assessing social media use would be valuable.

On the during COVID-19 survey, rates of benefits (13/20, 65%) and hazardous use (14/20, 70%) were similar to pre-COVID-19. Similar to pre-COVID, a minority of participants (4/20, 20%) indicated they routinely ask patients about social media use.

Among the 15 participants with data at both times points, there was a nonsignificant increase in the likelihood of asking about social media use (2/15, 13% vs 4/15, 27%, for pre- and during COVID-19, respectively; McNemar χ^2^_1_=0.25, *P*=.617, Cohen *d*=0.33). There was a nonsignificant increase in the likelihood of cases in which social media use was a benefit (10/15, 67% vs 11/15, 73%, for pre- and during COVID-19, respectively; McNemar χ^2^_1_=0.00, *P*=.999, Cohen *d*=0.14) and a nonsignificant decrease in the likelihood of cases in which social media use was hazardous (13/15, 87% vs. 11/15, 73%, for pre- and during COVID-19, respectively; McNemar χ^2^_1_=0.25, *P*=.617, Cohen *d*=–0.33).

## Discussion

We initially set out to examine if psychiatry residents ask patients about social media use as part of a quality improvement project. Subsequently, the COVID-19 pandemic and its impact on social media use at the population level motivated us to assess whether the psychiatric interview was modified accordingly. At both time points, survey responses suggest that social media use is rarely included by trainees during psychiatry interviews, despite the fact that respondents recognized the clinical relevance of social media use and were able to identify clinical cases of both benefit and harm. Although nonsignificant, there was a numeric increase in the likelihood of assessing social media usage during COVID-19, potentially in response to increased social isolation occurring in the population [13,15]. Not surprisingly, this small-magnitude effect (*d*=0.33) was not statistically significant in our sample of 15. Despite the possibility that rates may have increased, the fact that the vast majority of clinicians failed to assess social media usage even amidst COVID-19 highlights a potential gap in training.

There are important methodological limitations in this study. Consistent with a quality improvement project, we conducted no a priori sample size planning. Ultimately, the sample size was small, and analyses were underpowered to detect small or even moderate magnitude effects. Initially designed as a needs assessment, the survey design prioritized brevity and sacrificed complexity. Only a single residency was evaluated, thus limiting generalizability. Lacking a during COVID-19 assessment only group, we are unable to rule out the possibility that follow-up responses were influenced by repeated testing rather than COVID-19.

Future areas of study include querying faculty assessment and teaching practices, extending the survey to other residencies, evaluating barriers to assessing social media use, and investigating whether residents feel equipped to respond to higher-risk behavior or information they gather as a result of assessing for social media use. More broadly, future studies could evaluate residents’ comfort adapting the general interview to different sociocultural contexts and changes.

These limitations notwithstanding, we believe these preliminary data highlight an important training gap in the assessment of social media utilization during the psychiatric interview and areas for future study and improvement.

## Appendix

Multimedia Appendix 1 Survey items and responses.
