# Supplementary material for: Rates of Assessment of Social Media Use in Psychiatric Interviews Prior to and During COVID-19: Needs Assessment Survey
Source: JMIR Med Educ. 2021 Sep 14;7(3):e28495. doi: 10.2196/28495 (PMC8447920; doi:10.2196/28495)
Supplement: Multimedia Appendix 1 [file mededu_v7i3e28495_app1.docx]

**Supplemental Materials Table 1.** Pre- and post-COVID-19 survey and responses.

| Survey 1  N=21 |  | | | Survey 2  N=20 |  | |
| --- | --- | --- | --- | --- | --- | --- |
| Item 1. How would you rate your knowledge about social media and mental health? | Very familiar | | 9.5% (2/21) | NA |  | |
|  | Somewhat familiar | | 81% (17/21) |  |  |  |
|  | Not familiar: | | 9.5 % (2/21) |  |  |  |
| Item 2. Do you routinely ask patients about their use of social media? | Yes | | 9.5 % (2/21) | 1. Since March 21 have your routinely asked patients about their use of social media? | Yes | 20% (4/20) |
|  | No | | 85.7% (18/21) |  | No | 80% (16/20) |
|  | Inpatient Only | | 4.8% (1/21) |  | Inpatient Only | 0 |
|  | Outpatient Only: | | 0 |  | Outpatient Only: | 0 |
| Item 3. Have you encountered clinical cases where you considered the patient's use of social media to be of benefit? | Yes | | 61.9% (13/21) | 2. Since March 21, have you encountered clinical cases where you considered the patient's use of social media to be of benefit? | Yes | 65% (13/20) |
|  | No | | 38.1% (8/21) |  | No | 35% (7/20) |
| Item 4. Have you encountered clinical cases where you considered the patient’s use of social media to be hazardous? | Yes | 81% (17/21) | | 3. Since March 21, have you encountered clinical cases where you considered the patient's use of social media to be hazardous? | Yes | 70% (14/20) |
|  | No | 19% (4/21) | |  | No | 30% (6/20) |
| Item 5. Would a brief interview guide for assessing social media use be of value to you in your clinical practice? | Yes | 100% (21/21) | |  |  | |
|  | No | 0% | |  |  |  |
